# Fulminant Clostridioides difficile Infection During Repeated High-Dose Cytarabine Consolidation for Acute Myeloid Leukemia: A Fatal Case

**DOI:** 10.7759/cureus.102177

**Published:** 2026-01-23

**Authors:** Eisaku Iwanaga, Hirotomo Nakata, Rie Furuta, Kenji Tokunaga, Jun-Ichirou Yasunaga

**Affiliations:** 1 Department of Hematology, Rheumatology, and Infectious Diseases, Kumamoto University Hospital, Kumamoto, JPN

**Keywords:** acute myeloid leukemia, autopsy, chemotherapy-associated infection, clostridioides difficile, fulminant colitis, high-dose cytarabine, neutropenia, pseudomembranous colitis, recurrent infection, toxin b

## Abstract

Fulminant *Clostridioides difficile* infection (CDI) is a life-threatening complication during intensive chemotherapy. We report a 69-year-old woman with acute myeloid leukemia who had two CDI episodes of different severity following sequential high-dose cytarabine consolidation cycles. The initial episode was mild diarrhea during myelosuppression and was managed by oral metronidazole. Her diarrhea resolved by day 20, and she was discharged on day 24. She started her second cycle of consolidation 41 days after the first cycle with the same antibiotic prophylaxis. On day 17, she developed watery diarrhea and abdominal pain. Oral metronidazole was started for suspected recurrence. However, she rapidly developed severe metabolic acidosis and septic shock, requiring mechanical ventilation, vasopressors, and continuous hemodiafiltration. Computed tomography showed thickened colonic walls and ascites. The stool PCR was positive for toxin B and negative for hypervirulent strain markers. The aggressive therapy with intravenous metronidazole and nasogastric vancomycin failed, and she died within a few days. The autopsy revealed diffuse pseudomembranous colitis. This case illustrates the unpredictable severity of recurrent CDI during intensive chemotherapy. It highlights the critical importance of guideline-adherent antimicrobial therapy, particularly the use of fidaxomicin or vancomycin rather than metronidazole in high-risk patients.

## Introduction

*Clostridioides difficile* infection (CDI) is a common cause of antibiotic-associated colitis and is often observed among hospitalized and immunocompromised patients [1]. Despite the majority of the cases being responsive to proper antimicrobial treatment, fulminant CDI, which is associated with toxic megacolon, septic shock, and multiple organ failure, is still life-threatening [2,3]. Hematologic malignancy patients are also more susceptible to CDI due to intensive chemotherapy, prolonged neutropenia, and exposure to broad-spectrum antibiotics [4]. The prevention of the recurrence of CDI is a significant problem in the high-risk groups.

CDI is usually manageable in patients with acute myeloid leukemia (AML), but sudden deterioration can be fatal. The causes of such a sudden decline remain poorly understood [4,5]. It is unclear why some patients experience dramatically different levels of disease severity despite identical chemotherapy protocols [5].

We report an autopsy-confirmed case of fulminant CDI that occurred during the second cycle of high-dose cytarabine consolidation for AML, while the first cycle produced only a mild, treatment-responsive episode. This case highlights the unpredictable nature of recurrent CDI during intensive chemotherapy and underscores the importance of appropriate antimicrobial selection.

## Case presentation

A 69-year-old woman was diagnosed with AML harboring an NPM1 mutation and achieved complete remission after standard induction chemotherapy. She received her initial high-dose cytarabine consolidation with prophylaxis of levofloxacin, posaconazole, and a proton pump inhibitor. She had watery diarrhea during the myelosuppressive phase. CDI was diagnosed based on positive glutamate dehydrogenase (GDH) antigen and toxin A/B assays in stool. She was treated with oral metronidazole 500 mg every eight hours, showed prompt clinical improvement, and was discharged.

She received a second course of consolidation therapy using the same chemotherapy regimen and prophylaxis 41 days after the first cycle (18 days after discharge). Baseline laboratory values before her second consolidation cycle showed moderate inflammation (Table 1). On day 15, she developed febrile neutropenia, and cefepime was started. On day 16, she developed intermittent epigastric pain. On day 17, she developed frequent watery diarrhea. Given her history of CDI, recurrence was suspected. A rapid enzyme immunoassay detected GDH antigen and toxins A/B (approximately five to six weeks after the initial positive toxin assay, consistent with the commonly used two-to-eight-week window for recurrent CDI). Therefore, oral metronidazole was started. However, her fever persisted, intravenous teicoplanin was added, and cefepime was escalated to meropenem.

**Table 1:** Laboratory findings at baseline (day -1) and fulminant stage (day 18) “–” denotes tests that were not obtained at that time point. WBC: white blood cell count, Hb: hemoglobin, Plt: platelet count, Alb: albumin, T-Bil: total bilirubin, crea: creatinine, CRP: C-reactive protein, PT: prothrombin time activity, APTT: activated partial thromboplastin time

Parameter	Day -1	Day 18 (8:30)	Day 18 (10:34)	Day 18 (16:17)	Unit	Reference range
WBC	9400	100	-	-	/µL	3,300-8,600
Neutrophils	83	8	-	-	%	42.4-75.0
Lymphocytes	7	67	-	-	%	18.2-47.7
Monocytes	10	25	-	-	%	3.3-9.0
Hb	6.5	11.3	-	-	g/dL	11.6-14.8
Plt	18 × 10^4^	2.8 × 10^4^	-	-	/µL	15.8-34.8 × 10^4^
Alb	3.2	2.1	-	-	g/dL	4.1-5.1
T-Bil	0.5	1.1	-	-	mg/dL	0.4-1.5
Crea	0.64	1.28	-	-	mg/dL	0.46-0.79
CRP	3.93	38.9	-	-	mg/dL	0.00-0.14
PT	-	60	-	-	%	80-120
APTT	-	42.5	-	-	s	24.0-39.0
Fibrinogen	-	>700	-	-	mg/dL	200-400
D-dimer	-	5.5	-	-	µg/mL	<1.0
Venous pH	-	-	7.432	7.255	-	7.33-7.43
Venous pCO2	-	-	28.0	19.2	mmHg	40-50
Venous HCO₃⁻	-	-	18.3	8.3	mmol/L	22-29
Venous lactate	-	-	2.82	11.5	mmol/L	0.5-2.2

**Figure 1:**
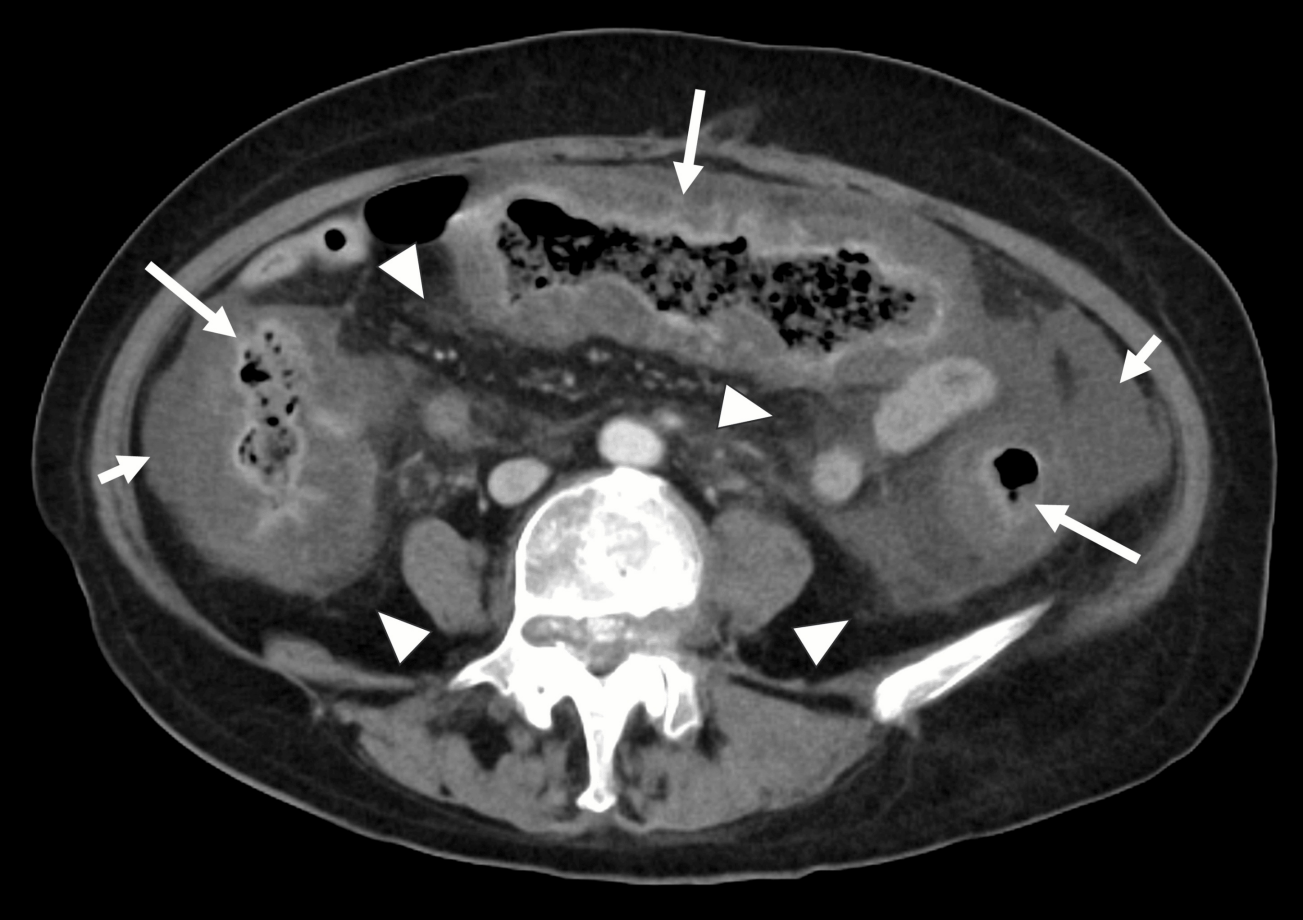
Contrast-enhanced abdominal CT on day 18 revealed marked edematous wall thickening from the rectum to the ascending colon (long arrows), with pericolonic fat stranding (arrowheads) and ascites (short arrows). No perforation or abscess formation was identified. Mucosal enhancement was preserved CT: computed tomography

Her abdominal pain deteriorated rapidly, despite treatment. On day 18 morning, she experienced severe, diffuse abdominal pain. Physical examination showed abdominal distension and diminished bowel sounds with sharp tenderness and rebound tenderness in the epigastrium and lower abdomen without guarding. Abdominal CT with contrast enhancement revealed significant edematous thickening of the walls of the entire colon without bowel necrosis, perforation, or abscess (Figure 1). She experienced shock in the evening of day 18 (blood pressure 87/33 mmHg, heart rate 101 bpm, respiratory rate 44 breaths per minute, temperature 36.4°C, SpO₂ 98% on room air) and reduced consciousness (Glasgow Coma Scale 14: E3V5M6). Venous lactate increased from 2.82 mmol/L at 10:34 to 11.5 mmol/L at 16:17, accompanied by worsening metabolic acidosis (Table 1). Norepinephrine was initiated, but adequate hemodynamic stabilization was not achieved. She was endotracheally intubated and transferred to the intensive care unit, where continuous hemodiafiltration was initiated. Vancomycin 500 mg every six hours was administered via nasogastric tube, and metronidazole was switched from oral to intravenous administration at 500 mg every eight hours. Subsequent stool testing showed *Clostridioides difficile* growth (10⁶ colony-forming units). Polymerase chain reaction detected toxin B but was negative for mutant tcdC and binary toxin genes.

**Figure 2:**
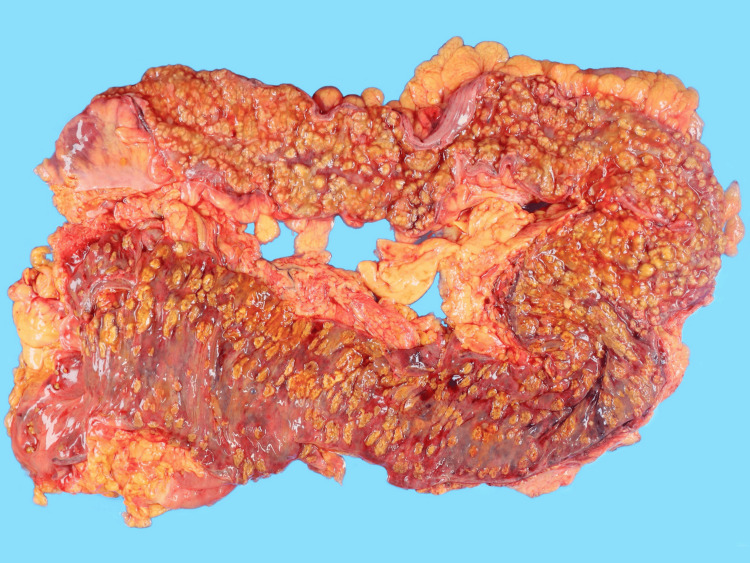
Multiple yellow-green flat elevations with accompanying mucosal erythema were observed extending from the colon to the rectum

**Figure 3:**
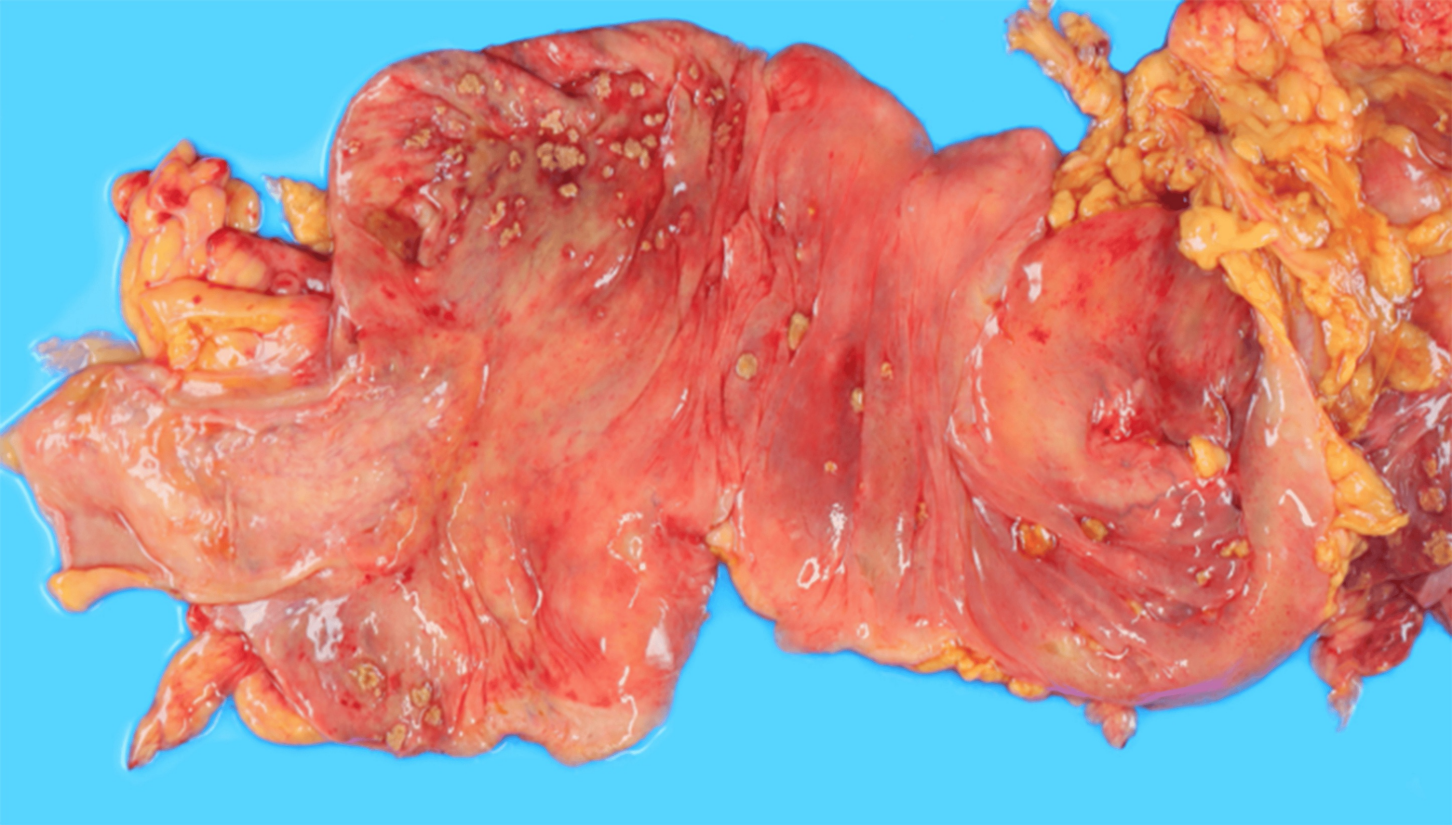
No pseudomembrane formation was observed in the ileum

**Figure 4:**
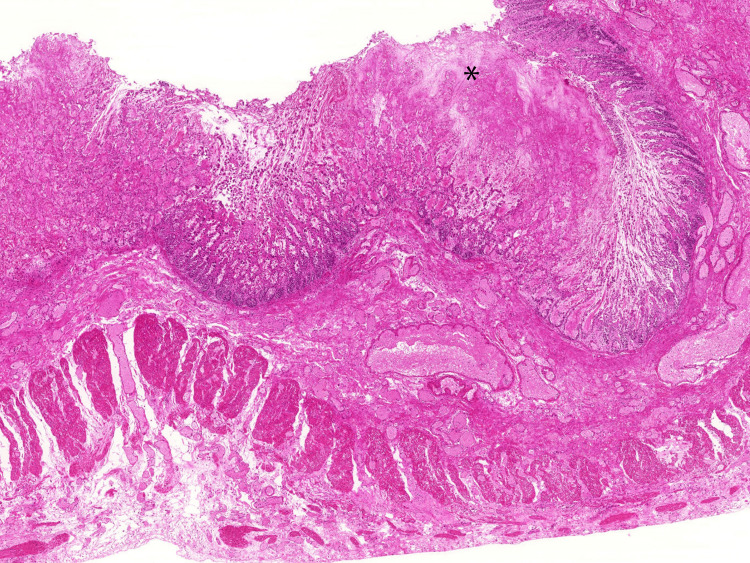
Hematoxylin and eosin-stained section showing mucosa-limited inflammation with eruptive pseudomembrane formation on the surface (asterisk) and marked transmural edema involving the mucosa, submucosa, muscularis propria, and serosa

**Figure 5:**
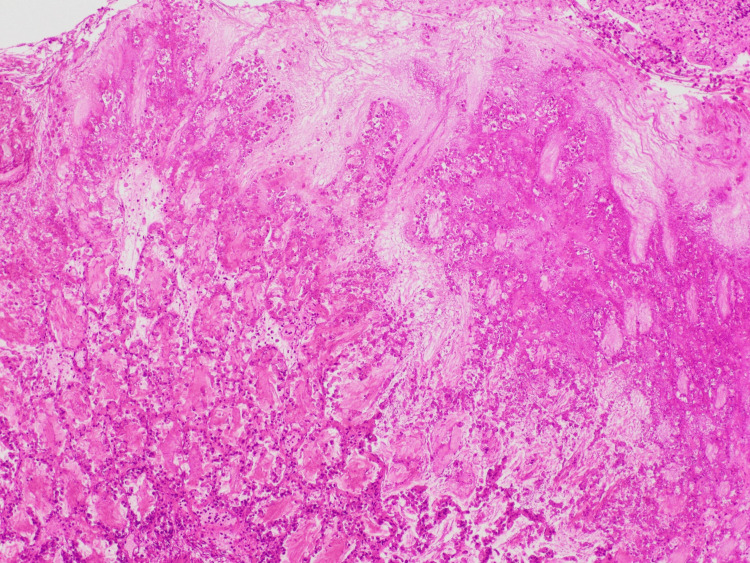
High-power view of the pseudomembrane indicated by the asterisk in Figure 4. The pseudomembrane was composed of mucus and fibrin, containing abundant neutrophils and necrotic debris. No pathogenic organisms suggestive of other infectious diseases were identified

By day 19, neutrophil recovery was observed; however, systemic perfusion did not improve, and she progressed to multiple organ failure and died on day 21. Autopsy showed that there were diffuse yellow-green flat elevations and inflammatory exudates that extended through the colon to the rectum, and the ileum was spared (Figures 2-3). Histopathological examination showed characteristic pseudomembrane formation on the colonic mucosal surface with marked transmural edema consistent with fulminant pseudomembranous colitis (Figures 4-5).

## Discussion

The case shows an extreme increase in the severity of CDI between two identical rounds of consolidation chemotherapy. There are several possible causes of the fulminant presentation in the second cycle: cumulative mucosal toxicity from two high-dose cytarabine cycles [6], the patient's age (69 years), hypoalbuminemia (3.2→2.1 g/dL), and neutropenia (100/μL). More importantly, the metronidazole selection in both episodes was not optimal. Recent Infectious Diseases Society of America (IDSA)/Society for Healthcare Epidemiology of America (SHEA) guidelines (2021) recommend fidaxomicin or vancomycin as first-line treatment, replacing metronidazole due to its lower efficacy and increased recurrence rates [7]. Fidaxomicin would have been a better choice in this high-risk patient undergoing intensive myelosuppressive chemotherapy to maximize the eradication of *Clostridioides difficile*. In cases where CDI recurred, the use of metronidazole (orally) rather than switching to vancomycin and fidaxomicin was a missed opportunity. This suboptimal antimicrobial selection may have contributed to incomplete clearance and recurrent infections of increasing severity.

An important finding is the absence of markers for hypervirulent strains. Binary toxin and mutant tcdC are used as surrogate markers of hypervirulence to characterize epidemic strains of RT027 and RT078 in Western countries. The PCR of our patient's stool was negative for both markers, indicating that this was not an epidemic hypervirulent strain. This is consistent with Japanese epidemiology: marker-positive strains are rare in Japan, with non-hypervirulent strains (RT018, RT014, RT002, RT369, RT017) comprising over three-quarters of isolates and capable of causing fulminant CDI [8]. Our case demonstrates that non-hypervirulent strains can lead to severe outcomes when combined with intensive chemotherapy and suboptimal antimicrobial therapy.

Few similar cases have been reported. Yamamoto et al. reported fulminant CDI in an elderly patient with AML undergoing low-intensity therapy that responded to fidaxomicin [9]. Lee et al. described a young AML patient who was treated successfully with fecal microbiota transplantation (FMT) [10]. Carmeliet et al. reported a case of an AML patient who needed a colectomy [11]. In contrast, our patient had high-intensity consolidation, cumulative cytarabine-induced damage, severe neutropenia, advanced age, and suboptimal antimicrobial therapy, all contributing to the fatal outcome. Limitations in treatment were the unavailability of FMT under insurance coverage in Japan and surgical contraindication because of severe neutropenia. A CT scan and autopsy confirmed CDI as the leading cause, with no other source of infection.

For patients undergoing high-dose cytarabine consolidation, adherence to current guidelines is essential. Initial CDI should be treated with fidaxomicin (preferred) or vancomycin rather than metronidazole to achieve complete eradication and minimize the risk of recurrence [7]. Secondary prophylaxis with oral vancomycin 125 mg twice daily may be considered for patients with previous CDI receiving additional cycles of intensive chemotherapy [1]. In the case of fulminant CDI, a combination of vancomycin (orally or nasogastrically) with intravenous metronidazole is indicated [7]. FMT represents an important rescue option when available [12]. Clinicians should recognize that subsequent high-dose cytarabine cycles carry substantial risk and that alternative consolidation strategies may need to be considered, balancing the risk of infection against leukemia control.

## Conclusions

This case demonstrates that CDI during repeated high-dose cytarabine consolidation may recur with unpredictably severe manifestations. The use of metronidazole rather than guideline-recommended fidaxomicin or vancomycin may have contributed to incomplete eradication and recurrence with a fatal outcome. In patients receiving intensive myelosuppressive chemotherapy, adherence to current IDSA/SHEA guidelines is important: fidaxomicin or vancomycin is recommended for initial CDI, and secondary prophylaxis may be considered for subsequent chemotherapy cycles. Clinicians must recognize that prior mild CDI does not predict the severity of recurrence, and treatment decisions should be guided by evidence-based recommendations rather than initial disease severity. Autopsy confirmation of diffuse pseudomembranous colitis without hypervirulent strain markers underscores that fulminant CDI can occur with typical strains when combined with intensive chemotherapy and suboptimal antimicrobial selection.
